# Maternal Mosaicism Challenges in Non‐Invasive Prenatal Diagnosis

**DOI:** 10.1002/pd.6868

**Published:** 2025-08-13

**Authors:** Margot Comel, Marina Lamairia, Odile Boute, Camille Cenni, Anne Bergougnoux, Mireille Cossée, Michel Koenig, Luke Mansard, Marie‐Claire Vincent

**Affiliations:** ^1^ Institut Universitaire de Recherche Clinique Montpellier Occitanie France; ^2^ INSERM U1046 Montpellier Occitanie France; ^3^ Centre Hospitalier Universitaire de Lille Clinique de Génétique Guy Fontaine Lille Hauts‐de‐France France; ^4^ Hôpital Universitaire Caremeau Nîmes Occitanie France

**Keywords:** cell free DNA, cell free fetal DNA, de novo variant exclusion, droplet digital PCR, maternal mosaicism, non‐invasive prenatal diagnosis

## Abstract

**Objective:**

To report the incidental detection of maternal somatic mosaicism during the development of exclusion‐based non‐invasive prenatal diagnosis for monogenic disorders (NIPD‐MD) initially indicated for apparently de novo pathogenic or likely pathogenic variants.

**Method:**

A droplet digital PCR (ddPCR)‐based exclusion NIPD_MD assay was developed for four couples, each with a prior pregnancy affected by a rare autosomal dominant or X‐linked condition due to a de novo pathogenic or likely pathogenic variant. Assays were designed to detect fetal‐specific variants in maternal plasma, with validation performed on parental and proband samples.

**Results:**

In four cases, maternal somatic mosaicism (3%–9%) among 70 personalized NIPD_MD (5.7%) was identified during assay validation, rendering NIPD_MD infeasible due to interference from maternal alleles. Each couple was informed of the elevated recurrence risk. Depending on their preferences, invasive prenatal testing or intensive ultrasound follow‐up was undertaken. Retrospective analysis of maternal sequencing data confirmed low‐level mosaicism that had been filtered out during routine analysis.

**Conclusion:**

These cases underscore a key limitation of exclusion NIPD_MD when maternal mosaicism is present. Its identification is essential for accurate recurrence risk estimation and genetic counseling. Sensitive detection methods, careful pre‐test evaluation, and transparent communication are critical to ensure informed reproductive decision‐making.

## Key Points

- What is already known about this topic?
  ◦ Non‐invasive prenatal diagnosis (NIPD) using digital droplet PCR (ddPCR) on maternal blood is a popular alternative to invasive investigations. However, this test excludes alterations inherited from the mother.
- What does this study add?
  ◦ We report four cases of maternal mosaicism identified during the validation of a test in an NIPD context. Maternal mosaicism represents 5.7% of our tests to date, increasing the risk of recurrence and ruling out the use of a ddPCR test for NIPD.

## 1 Introduction

Genetic mosaicism is defined as the presence of two or more cell lines in an individual, each bearing a distinct genotype, which arises from variants occurring after fertilization. Germline and somatic variations are inextricably linked and together shape human traits and disease risks. Germline variants are present from the moment of conception, but they vary between individuals and accumulate over generations. In contrast, somatic variations accumulate throughout an individual’s lifespan in a mosaic manner due to the combined influence of intrinsic and extrinsic sources of variations and selection pressures exerted on cells [1].

Both germline and somatic variants contribute to the development of human disease. When a pathogenic or likely pathogenic (P/LP) variant is identified by genetic analysis, parental testing is performed to ascertain the mode of inheritance and risk of recurrence. A de novo variant is defined as a variant identified in a proband but not inherited from his biological parents. The recurrence risk of a phenotype in this context is theoretically low, commonly estimated between 1% and 2% [2]. However, recent studies have highlighted that gonosomal mosaicism in parents accounts for 3%–29% of cases in various developmental disorder cohorts [3]. Furthermore, parental mosaicism is often underdiagnosed in routine settings, especially at low allele frequencies (< 5%), due to limitations in the sensitivity of traditional methods such as Sanger sequencing [4, 5].

Considering the recurrence risk of a rare and particularly serious disease, prenatal diagnosis (PND) can be proposed for determination of the fetus genotype. To limit the risks of miscarriages associated with current invasive PND procedures (chorionic villus sampling or amniocentesis), a personalized medicine‐based protocol for non‐invasive prenatal diagnosis (NIPD) of monogenic disorders (MD) has been developed relying on the detection of paternal or de novo P/LP variant in maternal blood using droplet digital PCR (ddPCR) [6].

NIPD_MD is based on the widespread use of cell‐free DNA from maternal apoptotic cells and from the trophoblasts, both contained in maternal blood. The coexistence and predominance of similar DNA sequences of maternal origin with fetal sequences make the biological analysis of circulating free DNA a complex undertaking. To date, NIPD_MD is limited to the detection of fetal‐specific sequences that are absent or different from the maternal genome referred as exclusion NIPD_MD. This non‐invasive approach exhibits the same degree of specificity, sensitivity, and turnaround time as invasive methods. Moreover, several studies suggest that the non‐invasive approach is the preferred option among patients and healthcare professionals when available as it considerably reduces parental anxiety [7]. For several years now, NIPD_MD has been commonly offered in France by laboratories belonging to the French NIPD_MD network. It is particularly suited to de novo P/LP variant recurrence risks although the development of NIPD_MD usually involves the development of targeted tests specific to each P/LP variant. In this study, we report incidental findings of maternal mosaicism detected during routine NIPD_MD by ddPCR for an a priori de novo variant.

## 2 Material and Methods

### 2.1 ddPCR

**FIGURE 1:**
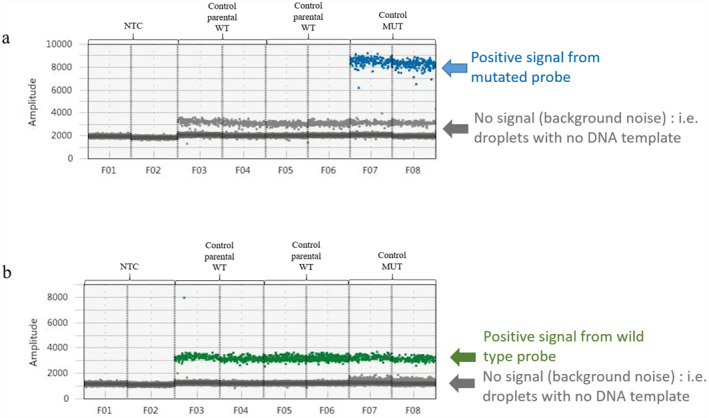
Droplet digital PCR results for an exclusion analysis of a de novo pathogenic variant. The *Y*‐axis corresponds to the fluorescence amplitude for droplets. (a) Blue dots are droplets containing FAM‐labeled fluorescent probe only, detecting the mutant allele. We can see that only the mutated control (probably a fetus) carries this variation (wells F07‐F08). There are no blue dots in either parent (wells F03‐F04 and F05‐F06). The line of gray dots represents negative droplets not carrying the mutation. (b) Green dots are droplets containing HEX‐labeled fluorescence probe only, detecting the wild allele. We can see that the proband (wells F07‐F08) and both its parents (wells F03‐F04 and F05‐F06) carry the wild allele. The line of gray dots represents negative droplets not carrying the mutation. MUT, mutated; NTC, no template control; WT, wild type.

Our laboratory specializes in developing targeted variants previously identified as responsible for monogenic pathologies. A specific exclusion NIPD_MD was developed for each variant using a ddPCR technique. The assay development was conducted in accordance with the previously described protocol by Gruber et al. on a Bio‐Rad Droplet Digital Platform (AutoDG instrument, C1000 thermocycler, and QX200 reader; Hercules, CA, USA) [8]. The design of the assay and the use of appropriate controls ensure the generation of robust and tailored tests. In particular, it is essential to consider the signal‐to‐noise limitation and to initially ascertain the performance of the assay, including its specificity, sensitivity, and linearity. In the case of de novo P/LP variant, the technique was developed using DNA samples from both partners in the couple and from the individual carrying the P/LP variant. The parents’ DNA serves as a reference point for a healthy control, exhibiting a positive signal for the HEX‐labeled fluorescence probe exclusively, thereby detecting the wild allele. Conversely, the DNA of the proband or affected fetus is employed as a positive control, exhibiting a positive signal for the FAM‐labeled fluorescence probe, which detects the mutant allele (see Figure 1a,b). Subsequently, the data obtained from ddPCR were analyzed using QuantaSoft Analysis Pro Software (Bio‐Rad, Hercules, CA, USA). The allelic fraction of a mosaic variant in a given DNA sample is calculated as the number of droplets containing a positive signal from the FAM‐labeled fluorescence probe (detecting the mutant allele) divided by the number of droplets containing a positive signal from the HEX‐labeled fluorescence probe (detecting the wild allele).

### 2.2 Ethical Permission

At the time of sample collection, written informed consent was provided by each family. The aforementioned tests were conducted in accordance with the ethical standards set forth in the Declaration of Helsinki.

## 3 Results

The following four cases of NIPD_MD exclusion development are presented for consideration and analysis. The following cases were received between August 2022 and March 2024 for four unrelated couples at risk of transmitting a monogenic disease to their offspring. These cases involved four distinct genes (*ARID1B*, *SMC1A*, *SOX2,* and *COL1A1*), with de novo P/LP variant, identified during a previous pregnancy with ultrasound abnormalities and resulting in medical termination of pregnancy (MTP). Given the low recurrence risk of de novo P/LP variant linked to germinal mosaicism, these four couples were optimal candidates for an exclusion NIPD_MD. The identification of maternal somatic mosaicism (with a rate of 3%–9%) during the design validation permits to inform on the high risk of recurrence and to precise the inability to perform NIPD_MD for these families.

**FIGURE 2:**
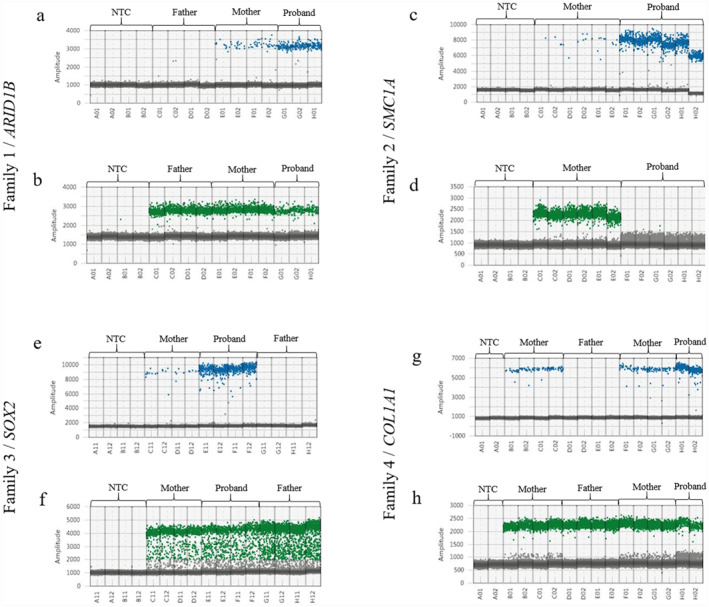
Droplet digital PCR results for an exclusion analysis of a pathogenic or likely pathogenic variant apparently identified as de novo by high throughput sequencing. Blue dots are droplets containing FAM‐labeled fluorescent probe only, detecting the mutant allele (a, c, e, and g). Green dots are droplets containing HEX‐labeled fluorescence probe only, detecting the wild allele (b, d, f, and h). The *Y*‐axis corresponds to the fluorescence amplitude for droplets. Two‐dimensional scatter plots showing the pathogen variant's detection in the four replicates in the probands' nuclear DNA, in a smaller allelic fraction in the mothers' DNA, its absence in the fathers' DNA and in a non‐template control (NTC). The low‐level pathogenic mutant allele dots in the mothers' DNA indicate a maternal somatic variant mosaicism. In *Family 1*, (a) blue dots are droplets containing FAM‐labeled fluorescent probe only, detecting the mutant allele. We can see that the proband clearly carries the *ARID1B* variation (wells G01‐G02‐H01). But we can also see positive droplets in the mother (wells E01‐E02‐F01‐F02). There are no blue dots in the father (wells C01‐C02‐D01‐D02). The line of gray dots represents negative droplets not carrying the mutation. (b) Green dots are droplets containing HEX‐labeled fluorescence probe only, detecting the wild allele. We can see that the proband (wells G01‐G02‐H01) and both parents (wells C01‐C02‐D01‐D02 in the father and wells E01‐E02‐F01‐F02 in the mother) carry the wild allele. The line of gray dots represents negative droplets not carrying the mutation. In *Family 2*, (c) blue dots are droplets containing only FAM‐labeled fluorescent probes, detecting the mutant allele. We can see that the proband clearly carries the *SMC1A* variation (wells F01‐F02‐G01‐G02‐H01‐H02). But we can also see positive droplets in the mother (wells C01‐C02‐D01‐D02‐E01‐E02). The line of gray dots represents negative droplets not carrying the mutation. (d) Green dots are droplets containing HEX‐labeled fluorescence probe only, detecting the wild allele. We can see that the proband (wells F01‐F02‐G01‐G02‐H01‐H02) does not carry this allele. The mother (wells C01‐C02‐D01‐D02‐E01‐E02) carried the wild allele. The line of gray dots represents negative droplets not carrying the mutation. In *Family 3*, (e) blue dots are droplets containing FAM‐labeled fluorescent probe only, detecting the mutant allele. We can see that the proband clearly carries the *SOX2* variation (wells E11‐E12‐F11‐F12). But we can also see positive droplets in the mother (wells C11‐C12‐D11‐D12). There are no blue dots in the father (wells G11‐G12‐H11‐H12). The line of gray dots represents negative droplets not carrying the mutation. (f) Green dots are droplets containing HEX‐labeled fluorescence probe only, detecting the wild allele. We can see that the proband (wells E11‐E12‐F11‐F12) and both parents (wells C11‐C12‐D11‐D12 in the mother and wells G11‐G12‐H11‐H12 in the father) carry the wild allele. The line of gray dots represents negative droplets not carrying the mutation. In *Family 4*, (g) blue dots are droplets containing only FAM‐labeled fluorescent probes, detecting the mutant allele. We can see that the proband clearly carries the *COL1A1* variation (wells H01‐H02). But we can also see positive droplets in the mother (wells B01‐B02‐C01‐C02 and F01‐F02‐G01‐G02). There are no blue dots in the father (wells D01‐D02‐E01‐E02). The line of gray dots represents negative droplets not carrying the mutation. (h) Green dots are droplets containing HEX‐labeled fluorescence probe only, detecting the wild allele. We can see that the proband (wells H01‐H02) and both parents (wells B01‐B02‐C01‐C02 and F01‐F02‐G01‐G02 in the mother and wells D01‐D02‐E01‐E02 in the father) carry the wild allele. The line of gray dots represents negative droplets not carrying the mutation. NTC, no template control.

In *Family 1*, a second pregnancy ultrasound monitoring led to the discovery of a singleton infant with agenesis of the corpus callosum. A prenatal whole‐exome sequencing was then proposed and identified a de novo nonsense variant in the *ARID1B* gene (NM_001374828): c.6337C>T ‐ p.(Arg2113Ter), which was interpreted as pathogenic according to ACMG‐AMP guidelines [9, 10]. The pregnancy complicated with maternal proteinuria and ended with a MTP at 33 weeks' amenorrhea. The observed phenotype is consistent with a Coffin‐Siris syndrome (MIM# 135900). Given the desire for a third pregnancy, an NIPD_MD assay to exclude this *ARID1B* variant was proposed. During the assay validation, a maternal somatic mosaicism of 9% was identified, which precluded performing an NIPD_MD (see Figure 2a,b). Consequently, the couple underwent an invasive PND (trophoblast biopsy), which did not detect the *ARID1B* pathogenic variant (PV) in the fetus. The mother gave birth to a healthy infant female at full term. Following the identification of the maternal somatic mosaic by ddPCR, a review of the sequencing data revealed that 5% of the maternal reads carried the pathological variant. However, these data had been filtered out due to low variant allele frequency.

The second couple (*Family 2*) underwent a MTP during their first pregnancy due to the identification of multiple malformations in the fetus. Whole genome sequencing was then proposed for the fetus and its parents and revealed a de novo missense variant in the *SMC1A* gene (NM_006306): c.3362G>A ‐ p.(Arg1121His). This alteration was interpreted as pathogenic in accordance with ACMG‐AMP guidelines [9]. The observed phenotype is consistent with the clinical presentation of Cornelia de Lange syndrome type 2 (MIM# 300590). For their second pregnancy, the couple agreed to undergo an exclusion NIPD_MD to avoid recurrence. During assay development, somatic mosaicism of approximately 6% was identified in the mother sample, which is incompatible with the realization of a NIPD_MD (see Figure 2c,d). Subsequently, a trophoblast biopsy was performed, concluding the absence of the variant for the current pregnancy. A review of the mother's sequencing data revealed that only one out of 55 reads carried the PV. As the ddPCR assay and the molecular analysis were conducted on the same DNA sample, a verification of the results was requested on a new one to eliminate the possibility of contamination. The results of the ddPCR tests were identical, indicating a maternal somatic mosaicism at a rate of 6%.

The third couple (*Family 3*) underwent a MTP of their first pregnancy at 24 weeks' amenorrhea due to the presence of microphthalmia, esophageal atresia, and an arachnoid cyst in their fetus. Whole genome sequencing for the trio revealed a de novo frameshift variant in the *SOX2* gene (NM_003106): c.205_206del ‐ p.(Ser69GlyfsTer26). In accordance with the ACMG‐AMP recommendations, this variant has been interpreted as likely pathogenic [10]. The phenotype is consistent with a syndromic microphthalmia type 3 (MIM# 206900). For the following pregnancy, an NIPD_MD approach was proposed. Maternal somatic mosaicism of 3%–5% was identified during the validation process of the assay, leading to the exclusion of the NIPD_MD strategy (Figure 2e,f). The couple chose to pursue close ultrasound monitoring of the pregnancy, which revealed no anomalies and led to the birth of a healthy female infant.

For the fourth couple (*Family 4*), a second pregnancy was terminated at 23 weeks' amenorrhea due to the suspicion of osteogenesis imperfecta in the fetus. A constitutional bone disease gene panel was realized on a fetal sample and identified a de novo missense variant in the *COL1A1* gene (NM_000088): c.1759G>T ‐ p.(Gly587Cys). This alteration was interpreted as pathogenic following ACMG‐AMP guidelines [9]. The observed phenotype is consistent with osteogenesis imperfecta syndrome type 2 (MIM# 166210). The fourth couple additionally opted for an exclusion NIPD_MD. Maternal somatic mosaicism of approximately 6% was identified during the test development (see Figure 2g,h). An invasive PND option was then presented to the couple, which they declined. Close monitoring of the pregnancy was realized and showed no abnormalities. Subsequently, the mother gave birth at term to a healthy female infant. Sanger sequencing was performed for familial segregation of the variant, which typically has a mosaic detection limit of around 20% [11]. However, the review of the electropherogram pointed out a very small signal of PV on the maternal sample, which was difficult to distinguish from background noise.

## 4 Discussion

To our knowledge, this is the first clinical report to document fortuitously discovered maternal somatic variation using ddPCR during the development of an exclusion NIPD_MD. In these four cases, it was impossible to use the ddPCR strategy for NIPD_MD, which led to an invasive diagnostic strategy or intensive clinical follow‐up.

Since 2017, the Institut Universitaire de Recherche Clinique (IURC) has afforded exclusion NIPD_MD. Of the 70 personalized exclusion NIPD_MD tests developed, four maternal somatic mosaicisms were detected, representing 5.7%. It is presumed that the mosaicism was pre‐existing and not induced or modified by the prior pregnancy. For these families, the time period between the first pregnancy of the affected child and the development of the test was between 5 and 20 months. Circulating fetal DNA remains in the blood for between 48 and 72 h [12]. Our standard delay ensures that the circulating fetal DNA of the current pregnancy is not contaminated by that of the previous one.

Our findings are consistent with previous reports highlighting the clinical importance of detecting low‐level parental mosaicism in the context of NIPD [2, 4, 13]. In a recent cohort, somatic parental mosaicism was found in 7.6% of families during the development of NIPD protocols [4]. In some of these cases, the mosaicism had been missed by Sanger sequencing and only revealed through high‐sensitivity approaches such as NGS or ddPCR. A parallel can therefore be drawn with the case report presented in this article.

In France, PND and MTP are considered when a fetus has a risk of being affected with a severe condition acknowledged as incurable at the time of diagnosis. When available, the realization of non‐invasive prenatal testing reduces couples anxiety regarding the potential risks associated with invasive procedures and provides definitive results in 96% of cases without delaying pregnancy management [6]. Although the overall prevalence of monogenic disorders is relatively high, individual cases remain rare, making widespread clinical implementation of personalized testing difficult. Using maternal blood samples, the detection of fetal alteration in cell‐free fetal DNA is still challenging due to its low proportion compared to maternal DNA [14].

Familial segregation of variants of interest was performed using whole exome or genome sequencing in three of the four couples in this study. None of these analyses reported the maternal mosaicism due to low variant allele frequency in mother's blood. The ability of high‐throughput sequencing to detect mosaic variations is contingent on the fulfillment of two conditions. First, the test must achieve high coverage. Secondly, the somatic variation must be present in blood cells [15]. Brewer et al. recommends a minimum of 50X coverage for targeted parental testing. This approach enables the sensitive detection of low‐level mosaic variants while maintaining reasonable costs. If the variant is detected in only a few reads (low variant allele frequency) at the 50X minimum coverage, it is then possible to re‐analyze the parental sample at a higher coverage. This may confirm the likelihood of the low variant allele frequency representing true mosaicism [16]. Consequently, when an apparently de novo P/LP variant is identified in a family study, the use of sensitive technology for the detection of low‐level mosaic variations in parental samples is essential for the accurate estimation of recurrence risk [17]. Molecular analysis of the variant found in tissues other than blood can also help determine the risk of gonosomal mosaicism [3, 18]. Unfortunately, we were unable to perform these tests in our case series.

Specific genetic syndromes are described as associated with an increased likelihood of parental somatic mosaicism for example, the Cornelia de Lange syndrome, which is caused by the P/LP variant in multiple genes, including *SMC1A* and particularly *NIPBL* [19]. Regarding post‐zygotic mosaicism in patients, up to 13% prevalence has been observed, representing a relatively high percentage in comparison with other genetic diseases. To date, more than 30 cases have been reported. Furthermore, the observed tissue distribution of mosaic variants shows great variability [20]. With regard to the other genes presented here, only a limited number of documented cases of *ARID1B* gonosomal mosaicism have been described to date [21]. In contrast, a considerably higher number of cases have been reported for *SOX2* [22, 23] and for *COL1A1* [24, 25, 26].

While the majority of de novo P/LP variants arise in the paternal germline, maternally inherited mosaic variants are associated with a higher recurrence risk [13, 18].

In these four reported cases, the occurrence of maternal mosaicism can be considered an incidental finding in the context of NIPD. It is notable that consent forms for these procedures typically do not include information about this potential outcome. A study demonstrated that the majority of consent forms for prenatal diagnosis by DNA sequencing failed to acknowledge the potential for the detection of maternal genetic variation [27]. Nevertheless, the identification of these maternal mosaicisms has a beneficial impact on the subsequent management of couples. In the context of rare diseases, it is of paramount importance to reassess the risk of transmission. The existence of a parental mosaicism may give rise to prenatal or pre‐implantation diagnosis [28]. The degree of mosaicism may vary unexpectedly across different tissues, even within the same embryonic lineage. Consequently, individuals with mosaic variation are at an elevated risk of having offspring with an inherited condition relative to the general population.

It is difficult to assess effective coverage for ddPCR in this report as very few centers in France perform this technique. However, the detection threshold of ddPCR (typically below 1%) makes it an extremely sensitive technique for detecting somatic mosaicism, especially at extremely low allele frequencies. This sensitivity could be achieved with WES/WGS techniques with deep sequencing on the targeted sequences. This is particularly relevant given that recurrence risk has been shown to increase significantly with higher levels of gonasomal mosaicism, reaching up to 50% for VAF > 6% [13]. Our results reinforce the importance of integrating sensitive molecular techniques, such as deep WES/WGS and ddPCR, into clinical workflows, especially for families with a history of apparent de novo variants. Moreover, when paternal origin is suspected, the use of sperm DNA, rather than blood, has been shown to improve mosaicism detection and risk stratification [29, 30]. These parental mosaicisms require sensitive detection methods enabling accurate assessment of low‐level mosaicism, which is essential for reliable recurrence risk assessment and informed reproductive decision‐making.

## 5 Conclusion

As high throughput sequencing is increasingly performed for familial variant searching, it is becoming feasible to identify clinically relevant genes that are particularly susceptible to germline mosaicism. Subsequently, these genes may be subjected to analysis for mosaicism during the initial genetic testing of probands by a highly sensitive method, thereby ensuring that clinically relevant mosaic variants are not filtered out by bioinformatics pipelines. The four cases presented here serve to illustrate the issue of maternal somatic mosaicisms, which represent a significant limitation of NIPD_MD. It is imperative that couples who elect to pursue this approach to PND are provided with comprehensive and transparent information regarding its potential implications. It is of great importance that couples are informed about the limitations of NIPD_MD and that this information is provided to them in an educated manner during the preliminary genetic counseling consultation.

## Ethics Statement

The aforementioned tests were conducted in accordance with the ethical standards set forth in the Declaration of Helsinki.

## Consent

At the time of sample collection, written informed consent was provided by each family.

## Conflicts of Interest

The authors declare no conflicts of interest.

## Data Availability

The data underlying this article will be shared on reasonable request to the corresponding author.
